# Genomic profiling of colorectal cancer with isolated lung metastasis

**DOI:** 10.1186/s12935-020-01373-x

**Published:** 2020-07-01

**Authors:** Nan Zhang, Jiabo Di, Zaozao Wang, Pin Gao, Beihai Jiang, Xiangqian Su

**Affiliations:** Key Laboratory of Carcinogenesis and Translational Research (Ministry of Education), Department of Gastrointestinal Surgery IV, Peking University Cancer Hospital & Institute, 52 Fucheng Road, Haidian District, Beijing, 100142 China

**Keywords:** Colorectal cancer, Lung metastasis, Whole exome sequencing, Somatic single nucleotide variation, Somatic copy number alteration, Clonal evolution

## Abstract

**Background:**

Metastasis is a major cause of failed colorectal cancer (CRC) treatment. While lung metastasis (LM) is observed in 10–15% of patients with CRC, the genetic mechanisms that cause CRC to metastasize to the lung remain unclear.

**Methods:**

In this study, we employed whole exome sequencing (WES) of primary CRC tumors and matched isolated LM lesions to compare their genomic profiles. Comprehensive genomic analyses of five freshly frozen primary tumor lesions, five paired LM lesions, and matched non-cancerous tissues was achieved by WES.

**Results:**

An integrated analysis of somatic mutations, somatic copy number alterations, and clonal structures revealed that genomic alterations were present in primary and metastatic CRCs with various levels of discordance, indicating substantial levels of intertumor heterogeneity. Moreover, our results suggest that the founder clone of the primary tumor was responsible for the formation of the metastatic lesion. Additionally, only a few metastasis-specific mutations were identified, suggesting that LM-promoting mutations might be pre-existing in primary tumors.

**Conclusions:**

Primary and metastatic CRC show intertumor heterogeneity; however, both lesions were founded by the same clone. These results indicate that malignant clones contributing to disease progression should be identified during the genetic prognosis of cancer metastasis.

## Background

Colorectal cancer (CRC) is a leading cause of cancer-related death worldwide and the number of patients being diagnosed with CRC is growing in China [1, 2]. Metastasis is a major cause of failed colorectal cancer (CRC) treatment. Approximately 50% of CRC patients that undergo radical resection of the primary tumor go on to develop metastatic disease, with the most common metastatic sites being the liver and lung [3, 4]. Cancer metastasis to distant organs is thought to occur via lymphatic or vascular drainage; disseminated colon and upper-rectal tumor cells enter the portal vein and arrive at the liver. Thus, clinically, the liver is the most common site of distant metastases [5]. Clinically, CRC with isolated lung metastasis (LM) is less common and is observed in 10–15% of CRC patients [6]. Isolated LM is characterized by the absence of extrapulmonary metastasis [7]. Approximately 35% of CRC patients with LM develop isolated initial tumors, and patients with colon cancer have a lower incidence of initial LM than patients with rectal cancer have [7].

The specific molecules involved in CRC LM have been investigated. For example, NDRG1 plays an important role in MORC2-mediated CRC cell migration and invasion in vitro, and promotes LM of CRC cells in vivo [8]. Elevated FOXC1 expression is significantly associated with CRC metastasis, recurrence, and reduced survival [9]. In vitro, FOXC1 up-regulation enhances CRC invasion and LM, while its down-regulation has the opposite effects [9]. In a mouse model, SMAD4-deficient CRC cells were found to secrete CCL15, which can recruit CCR1+ tumor-associated neutrophils, resulting in metastasis to the lung [10]. PP4C expression is frequently increased in CRC, and it’s up-regulation is correlated with CRC cell proliferation, migration, and invasion in vitro, as well as tumor growth and LM in vivo. PPC4 promotes cell invasion by up-regulating MMP-2 and MMP-9 via Akt phosphorylation [11]. Through an integrated analysis of mutation, copy number, and gene expression data, Fang et al. [12] identified a JAZF1 mutation with a copy number gain in a primary colon tumor and its matched LM, suggesting its oncogenic potential in both the colon to the lung.

Metastatic progression from the primary tumor requires multiple factors, such as the accumulation of genetic and epigenetic changes, and the capacity to colonize distant organs [13]. Accordingly, genetic aberrations and underlying mechanisms can influence the frequency and biological characteristics of CRC LM. However, beyond the function of the above-mentioned molecules in CRC LM, the genetic mechanisms underlying CRC metastasis to the lung remain unclear. It is possible that studies based on a small number of genes may misinterpret the extent of genetic alterations involved in primary and metastatic tumors.

To overcome this, we performed whole exome sequencing (WES) of primary CRC tumors and matched LM pairs to compare their genomic profiles. Specifically, WES was performed on five freshly frozen primary tumor lesions, five paired lung metastatic lesions, and matched non-cancerous tissues. We analyzed the somatic mutations, copy number variation, and clonal evolution in these patients. Our results identified intertumor heterogeneity and suggest that the founder clone from the primary tumor also formed the metastatic lesion. These results indicate that malignant clones contributing to disease progression should be identified during the genetic prognosis of cancer metastasis.

## Materials and methods

### Tumor specimens

This study included five CRC patients synchronously or metachronously diagnosed with LM who underwent tumor resection at the Department of Gastrointestinal Surgery IV, Peking University Cancer Hospital & Institute. Written informed consent was obtained from all patients. Clinical sample use was approved by the Research Ethics Committee of Peking University Cancer Hospital & Institute, Beijing, China (No. 2019KT93). All surgically resected tumor tissues were freshly frozen and stored at − 80 °C. For each metastatic tumor pair, a matched normal non-cancerous colorectal tissue sample was used as a germline control.

### DNA extraction

Genomic DNA was extracted from frozen tissue with a standard phenol/chloroform extraction. Briefly, tissue samples were fully ground with liquid nitrogen and the nuclei were suspended in extraction buffer (1 M sodium chloride, 100 mM Tris, and 50 mM EDTA [pH 8.0]) containing 2% sodium dodecyl sulfate (SDS) and proteinase K (2 mg/ml final concentration). Suspended nuclei were incubated at 56 °C for 2 h and DNA was first extracted with phenol:chloroform:isoamyl alcohol (25:24:1 volume), then with chloroform:isoamyl alcohol (24:1 volume), and precipitated with 0.7 volumes of isopropyl alcohol at − 20 °C for 40 min. DNA precipitates were washed twice with ice-cold 80% ethanol, collected by centrifugation (12,000 rpm, 15 min, 4 °C), dried under vacuum, and resuspended in 100 μl of EB (10 mM Tris hydrochloride [pH 8.0]) (Qiagen, Hilden, Germany). DNA quantity and quality were assessed using a NanoDrop One (ND-ONE-W; Thermo Fisher Scientific Inc., Waltham, MA) and with 1% agarose gel electrophoresis.

### Whole exome sequencing

Exome enrichment was performed with the xGen Exome Research Panel v1.0 (Integrated DNA Technologies, Inc., Coralville, IA) according to the xGen hybridization capture of DNA libraries protocol for next generation sequencing (NGS) target enrichment. Sequencing was performed on a HiSeq X Ten system (Illumina, Inc., San Diego, CA) according to the manufacturer’s instructions.

### Sequence alignment

Data were preprocessed using fastp (Version: 0.19.5) [14]. Clean reads were generated with the following filtering steps: (1) adapter sequences were removed; (2) reads with five or more N (non-AGCT) bases were removed; (3) a 4-base sliding window was set and used to remove regions with an average base quality value of less than 20; and (4) reads shorter than 75 bp or with quality values of less than 15 were removed. Clean reads were aligned to the hg19 reference genome using Burrows–Wheeler Aligner (BWA) (Version 0.7-6a) [15]. The Sequence Alignment/Map (SAM) software SAMtools (Version 1.4) was used to align sequences and convert the data to Binary Alignment/Map (BAM) format. Redundant information and noise were removed using Picard (Version 2.18.25).

### Variant calling

The HaplotypeCaller module of the genome analysis toolkit GATK (Version 4.1.0.0) was used to recalibrate the base quality score for single nucleotide variations (SNVs) and insertion/deletions (indels). Somatic SNVs and somatic indels were called with MuTect (version 2.0) by comparing the sequencing reads of the tumor and matched normal tissue [16]. Functional annotation of mutation sites was performed using ANNOVAR (Version 14 Dec 2015) [17].

### Determination of significantly mutated genes (SMGs)

Significantly mutated genes (SMGs) include genes with a significantly higher mutation rate than the background mutation rate (BMR). With the somatic mutations of all tumor samples as background, MuSiC (Version 0.4.1-1) [18] was used to detect genes with significantly higher mutation rates than the BMR. The SMG test was performed with three methods, including the convolution test (CT), Fisher’s combined P-value test (FCPT), and the likelihood ratio test (LRT). Genes with a false discovery rate (FDR) ≤ 2 in any two of the three tests were considered SMGs. Pathway enrichment analysis of SMGs was performed using PathScan [19].

### Analysis of somatic copy number alteration (SCNA)

Control-FREEC (Version 11.3) [20] was used to detect SCNAs. GISTIC [21] (Version 2.0) was used to identify significant copy number gains and losses. Each variation was assigned a G value after considering the mutation frequency and variation extent. The FDR q-value was then calculated, and q-values ≤ 0.25 were considered significant copy number gains and losses.

### Analysis of tumor clonality and construction of phylogenetic trees

SCNAs, SNVs, and indels were combined to obtain the input for PyClone (Version 0.13.1) [22], which was used to estimate the clonal cell fraction and construct the clonal structures of primary and metastatic tumors. The Bayesian model and Dirichlet process clustering were applied for grouping and estimating cellular prevalence. Next, ClonEvol (Version 2017) [23] was applied for clonal ordering and clonal evolution visualization.

### Gene ontology (GO), Kyoto Encyclopedia of Genes and Genomes (KEGG), and protein–protein interaction (PPI) analyses for LM-specific mutations

For GO analysis, all protein coding genes were used as the background list and genes with LM-specific mutations were used as the candidate list. The significance of GO function sets in the selected gene lists was calculated by hypergeometric distribution test. GO terms with more than two corresponding genes in the three classifications were screened, and the top 30 GO term bar chart was generated using the top 10 terms based on − log10 P value.

The KEGG database was used for pathway analysis of LM-specific genes (combined with KEGG annotation results), and a hypergeometric distribution test was applied to calculate the significance of gene enrichment in each KEGG Pathway entry. Pathway entries with more than two corresponding genes were screened based on descending − log10 P values. Then the top 20 entries were used to generate the KEGG top 20 bubble chart.

Based on the annotation of species information in the STRING database or blast (e-value < 10^−10^) of gene sequences and protein sequences, gene correlation was obtained and interactions between selected genes were extracted. Based on this information, an interaction network map was generated using Cytoscape, an open source platform for visualizing complex networks.

### Data availability statement

The data that support the findings of this study are available from the corresponding author upon reasonable request.

## Results

### Genomic landscape of CRC patients with LM

WES was performed on 15 freshly frozen samples from five CRC patients with LM, including five primary tumor foci, five matched LM foci, and five non-cancerous colorectal tissue samples as germline controls. The clinicopathological characteristics of all patients are shown in Additional file 1: Table S1. Among the five patients, patients 372, 374, and 375 underwent oxaliplatin and capecitabine therapy after resection of the primary tumor, while patients 371 and 373 did not receive chemo-, radio-, or targeted-therapy. Patients 371, 372, and 374 had metachronous LM, whereas patients 373 and 375 had synchronous LM.

**Fig. 1:**
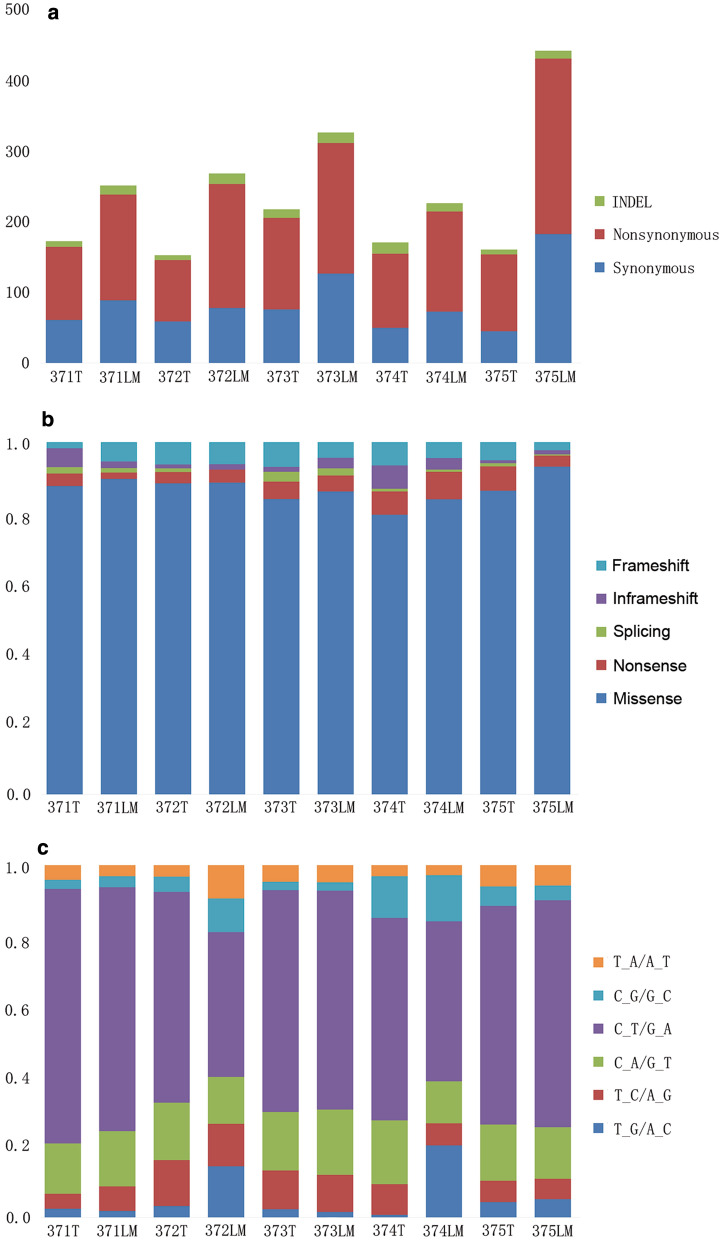
Mutations in CRC with LM. **a** Indel (green), nonsynonymous (red) and synonymous (blue) SNVs in the five CRC patients with LM. **b** For each patient, the relative fraction of five functional categories is shown. **c** Mutation spectrum of the five CRC patients with LM. Color codes represent the fraction of different base substitutions

After removing duplicates, we obtained an average target depth of 286× per sample (ranging from 214 to 475×, Additional file 2: Table S2). If a nucleotide substitution occurred in at least 10% of reads in at least one tumor lesion, it was considered to be a mutation. We identified 1421 non-synonymous and 841 synonymous somatic SNVs and 113 indels. Additional file 3: Table S3 shows the complete list of mutations for each patient. The number of different mutation types and functional consequences in each tumor are shown in Fig. 1a, b. Missense mutations were the dominant mutation category in both primary and metastatic tumors. Our results indicate that C > T transitions were the dominant mutation signature of both the primary tumor sites and metastases, accounting for 59.69% of all detected mutations, consistent with those reported for CRC [24, 25] (Fig. 1c).

### Mutation analysis reveals genomic heterogeneity in primary CRC with LM

**Fig. 2:**
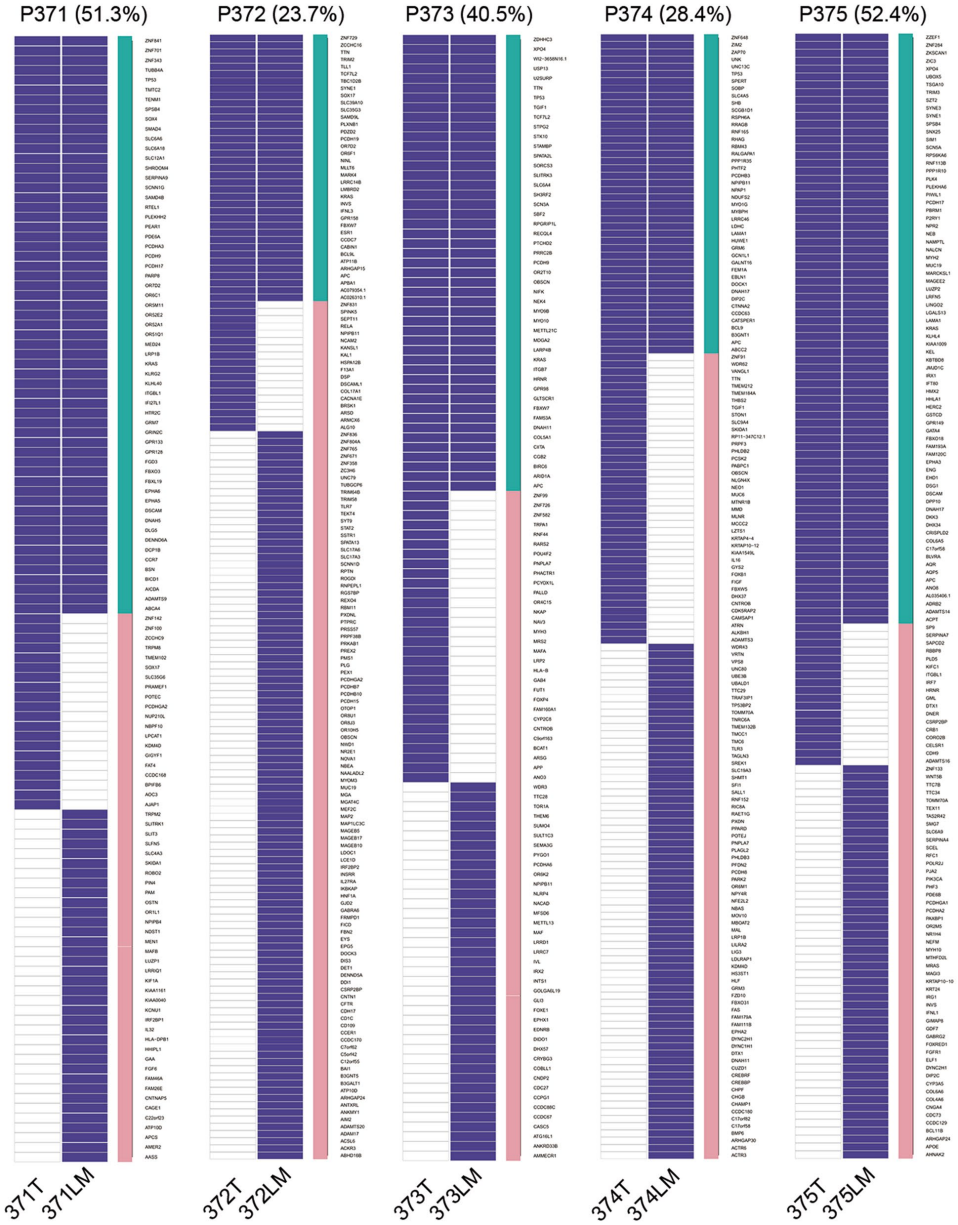
Distribution of somatic nonsynonymous mutations in CRC patients with LM. Heatmaps show the focal distribution of all nonsynonymous mutations. Presence (purple) or absence (white) of each mutation is marked for each tumor within one individual. Trunk (green) and lesion specific (pink) mutations are indicated. The percentage of trunk mutations in each patient is indicated on the top of the figure next to patient ID. Mutated genes are listed on the right of each heatmap

We next analyzed the distribution of non-synonymous mutations at the primary tumor sites and metastases in each patient (Fig. 2). WES identified an average of 144 (ranging from 87 to 249) non-synonymous mutations per sample. The average number of non-synonymous mutations in primary tumors and metastases was 107 and 180, respectively. Trunk mutations were defined as genes mutated in all lesions in a single patient. The percentages of trunk mutations ranged from 23.7 to 52.4%. These results indicate a varied mutational heterogeneity of primary tumors with metastatic lesions.

**Fig. 3:**
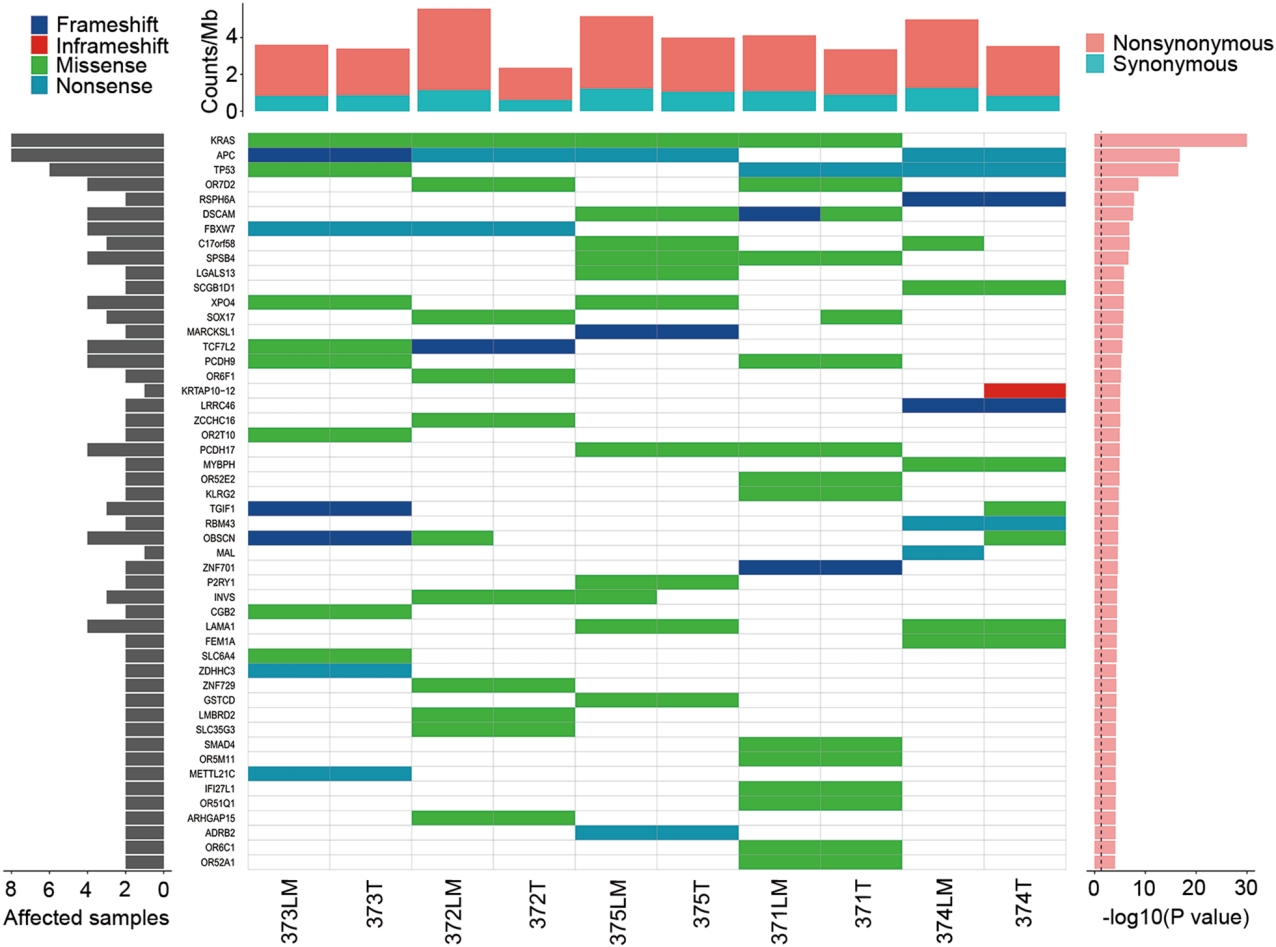
Significantly mutated genes (SMGs) in CRC patients with LM. The upper panel shows the gene mutation rate of each sample, calculated by number of mutations per megabase (Mb) of target sequence. Nonsynonymous (red) and synonymous mutations (green) were included. The central matrix shows SMGs and mutation types (represented by different colors as indicated in the upper left corner). Each column represents a single tumor lesion, and each row represents a gene. Patient ID is listed at the bottom of the figure. Blank grids indicate no mutations or no functional mutations in the corresponding gene of the sample. The grey bars on the left panel indicate the proportion of samples with mutated genes. The red bars on the right panel show SMGs ranked by − log10 P-value

We then determined the number of SMGs in primary and metastatic tumors. SMGs show a significantly higher mutation rate than the BMR when including multiple mutational mechanisms, such as coding indel, single nucleotide substitution, and splice site mutations [18]. We performed multiple statistical analyses, including CT, LRT, and FCPT, to determine P-values and FDR. A total of 145 SMGs were identified (Additional file 4: Table S4), the top 50 of which are shown in Fig. 3. KRAS, APC, TP53, and OR7D2 were the most highly mutated genes, with a FDR < 0.2 calculated by all methods. Given their important roles in CRC tumorigenesis and progression, it is not surprising that the cancer genes APC, TP53, and KRAS were the most prevalent SMGs [26]. However, the function of OR7D2 is currently unknown. Other frequently mutated genes which are often found in non-hypermutated CRCs [27], such as PIK3CA and NRAS, were rarely mutated. Furthermore, we analyzed the distribution of mutation sites in ubiquitous SMGs in tumor lesions from the same patient. Our results showed that mutations of all non-synonymous trunk SMGs were the same, indicating a common driver event in primary CRCs and their metastatic lesions, whereas lesion-specific somatic mutations are acquired during tumor development.

### Somatic copy number alteration (SCNA) analysis reveals chromosomal heterogeneity in primary CRC with LM

**Fig. 4:**
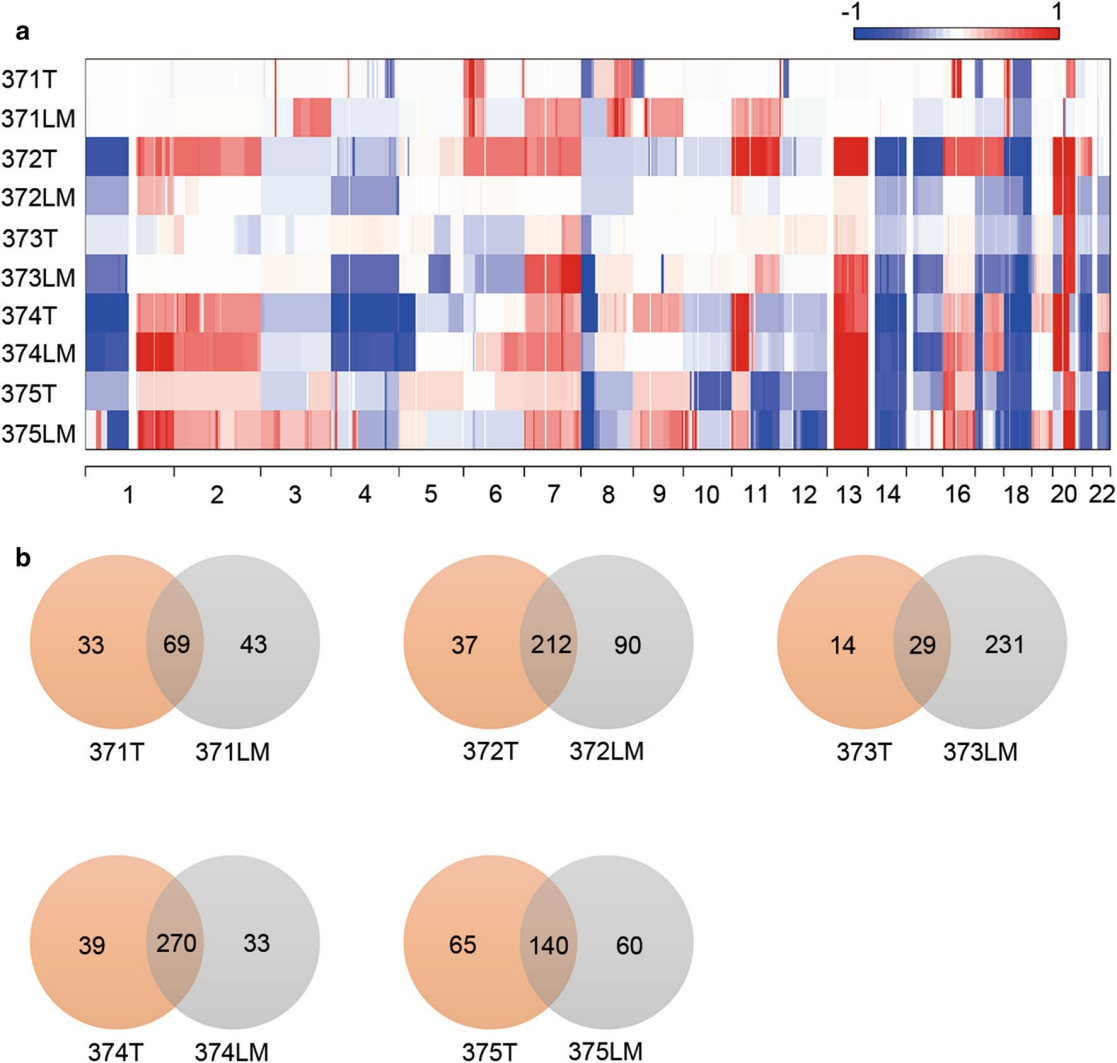
Somatic copy number alteration (SCNA) analysis of the five CRC patients with LM. **a** SCNA heatmaps of five CRC patients with LM. Sample names are labeled on the left side. The colors in the heatmaps represent copy number gain (red) and loss (blue). A gain was defined as a copy number at least one greater than ploidy, whereas a loss was defined as a copy number at least one less than ploidy. **b** Venn diagrams show the number of ubiquitous and lesion-specific SCNAs in each patient. Sample IDs are shown under each diagram

SCNA events occur early during tumorigenesis [28–30]. We assessed SCNAs to determine the role of chromosomal alternations in CRC with LM. Copy number gains and losses were identified in all lesions, indicating their important role in tumor progression and development. Segmented copy number calls derived from Control-FREEC and the affected driver genes are listed in Additional file 5: Table S5. SCNA analysis revealed disparate profiles within the patients (Fig. 4a). Some chromosomal aberrations were shared among lesions from the same patient shared; however, the average ubiquitous SCNA rate was 50.5%, ranging from 10.6 to 78.9% (Fig. 4b), indicating substantial intertumor chromosomal heterogeneity.

Further, GISTIC analysis (Additional file 6: Figure S1A) showed that 3p22.1, 19p13.3, 1q21.1, 16q24.3, 20q11.21, 11p15.5, 6p22.1, 11q12.1, and 5q35.3 were the most frequently amplified chromosomal regions. Gain of 20q, which is involved in the transformation of adenomas into carcinomas, has been reported in CRC [31]. In addition, amplification in 20q is associated with a worse prognosis of CRC [32]. The amplified genes in 20q belong to several signaling pathways that may be crucial in CRC development. For example, BCL2L1, located in 20q11.21, regulates the mitochondrial apoptosis pathway [33]. The most frequently deleted chromosomal regions include 4q35.2, 21q22.3, 1q21.2, 14q11.2, 22q13.33, 15q24.2, 1p21.3, 5q35.3, 10p15.3, 11q11, 5q22.2, and 5p12. Loss of 1p is associated with CRC invasion and poor prognosis [34, 35]. Deletions of 10p15.3-p14 may be correlated with poor prognosis of CRC [36]. Significant chromosomal gains and losses and the affected genes are listed in Additional file 7: Table S6.

Next, we compared the genes affected in recurrently altered chromosomal regions with the Cancer Gene Census database and identified the following high-risk genes: APC, ATP1A1, BCL10, CTNNB1, FAM46C, FUBP1, HLA-A, JAK1, NOTCH2, NRAS, RBM15, RPL5, TRIM33, and VAV1. High-risk genes includes genes documented in the KEGG pathways in cancer. KEGG analysis of these genes revealed that signaling pathways regulating the pluripotency of stem cells, T-cell receptor signaling pathway, B-cell receptor signaling pathway, and natural killer (NK) cell-mediated cytotoxicity were among the most significantly affected pathways. KEGG enrichment analysis is shown in Additional file 6: Figure S1B and Additional file 8: Table S7.

### Primary and metastatic CRCs exhibit different clonal structures

Tumors are often composed of several genotypically distinguishable cell populations, defined as clones. Clonal architecture may change to adapt to the tumor microenvironment and drug intervention used. Therefore, we next compared the clonal components of primary CRCs with their matched LMs (Additional file 9: Figure S2). Different clone structures of primary and metastatic tumors were observed, indicating variable genetic determinants of the phenotype, and different proliferation capacities. Different organ environments and drug interventions may also result in different clone structures of the primary and metastatic tumors. Accordingly, they may also have different chemotherapeutic responses.

### Phylogeny of CRC with LM

**Fig. 5:**
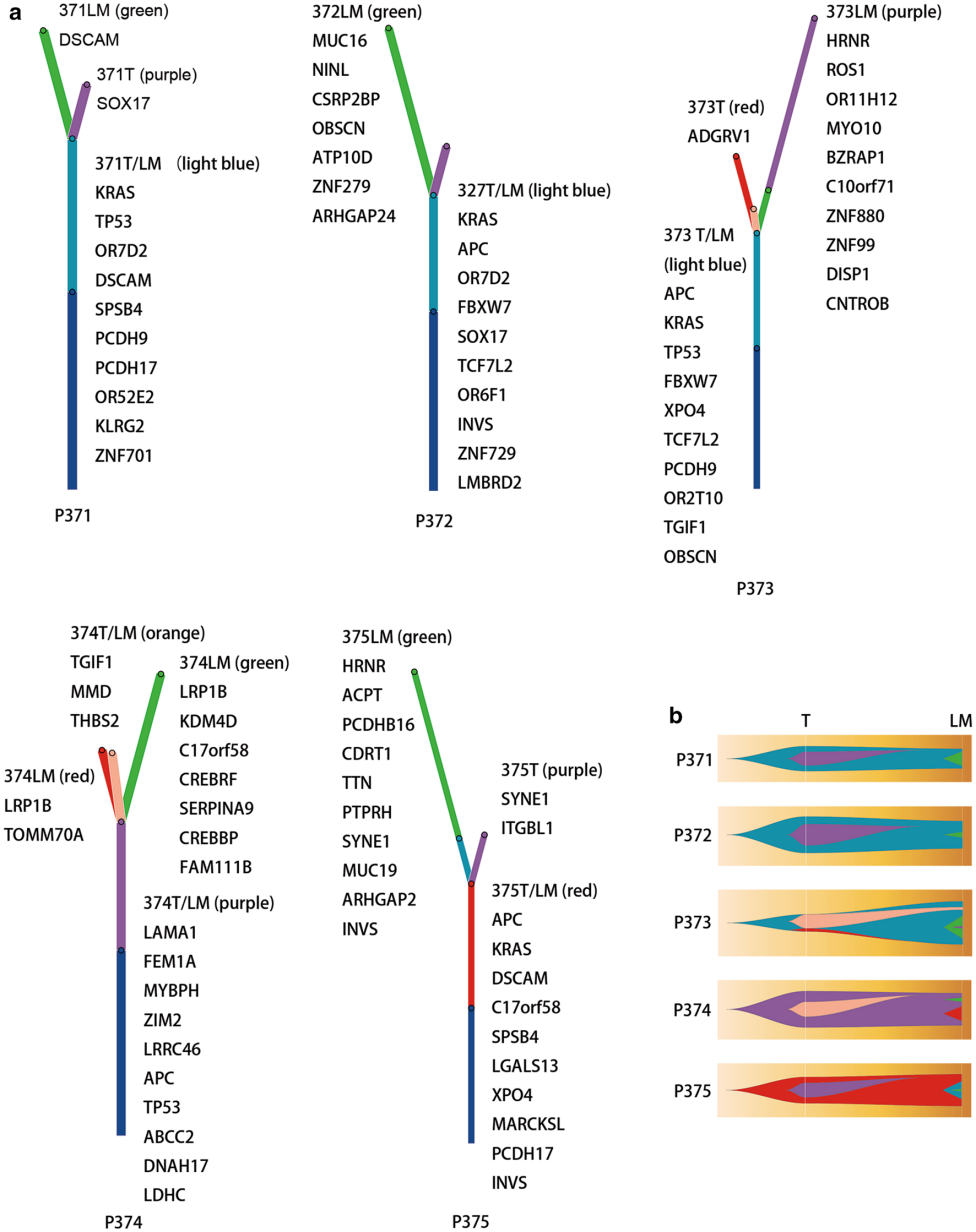
Clonal evolution of CRC LM. **a** Branch-based phylogenic trees for each patient. Branch-based clonal evolution trees are annotated with samples where the clones are present and driver events. **b** Fish plots inferred by ClonEvol showing an evolutionary model of CRC patients with LM

To further investigate the evolutionary process of CRC with LM, we performed phylogenetic reconstruction of WES data for each patient using ClonEvol. Branch-based trees represent clonal relationships and seeding patterns between samples, and the fish plots represent clonal dynamics over time (Fig. 5). The first notable observation was that the phylogenetic structure was similar across patients, and branched evolution is readily visualized. Based on evolution models, primary CRCs were founded by a single clone, and some new clone(s) were formed during tumor progression, some of which acquired metastatic potential. Upon metastasis, new clone(s) with growth advantages were generated. Clonal expansion of these new clones may lead to their dominance. The founding clone in the primary tumor also evolved into the metastatic clone. For patients 373 and 375, the metastatic tumors inherited genetically distinct subclones from the primary tumors, indicating a possible polyclonal seeding mechanism for metastasis (Fig. 5b). We identified potential driver mutations on the trees to reflect their time of acquisition; TP53, KRAS, and APC were frequently identified in the trunks (Fig. 5a).

### Profiling of LM-specific genetic alternations

**Fig. 6:**
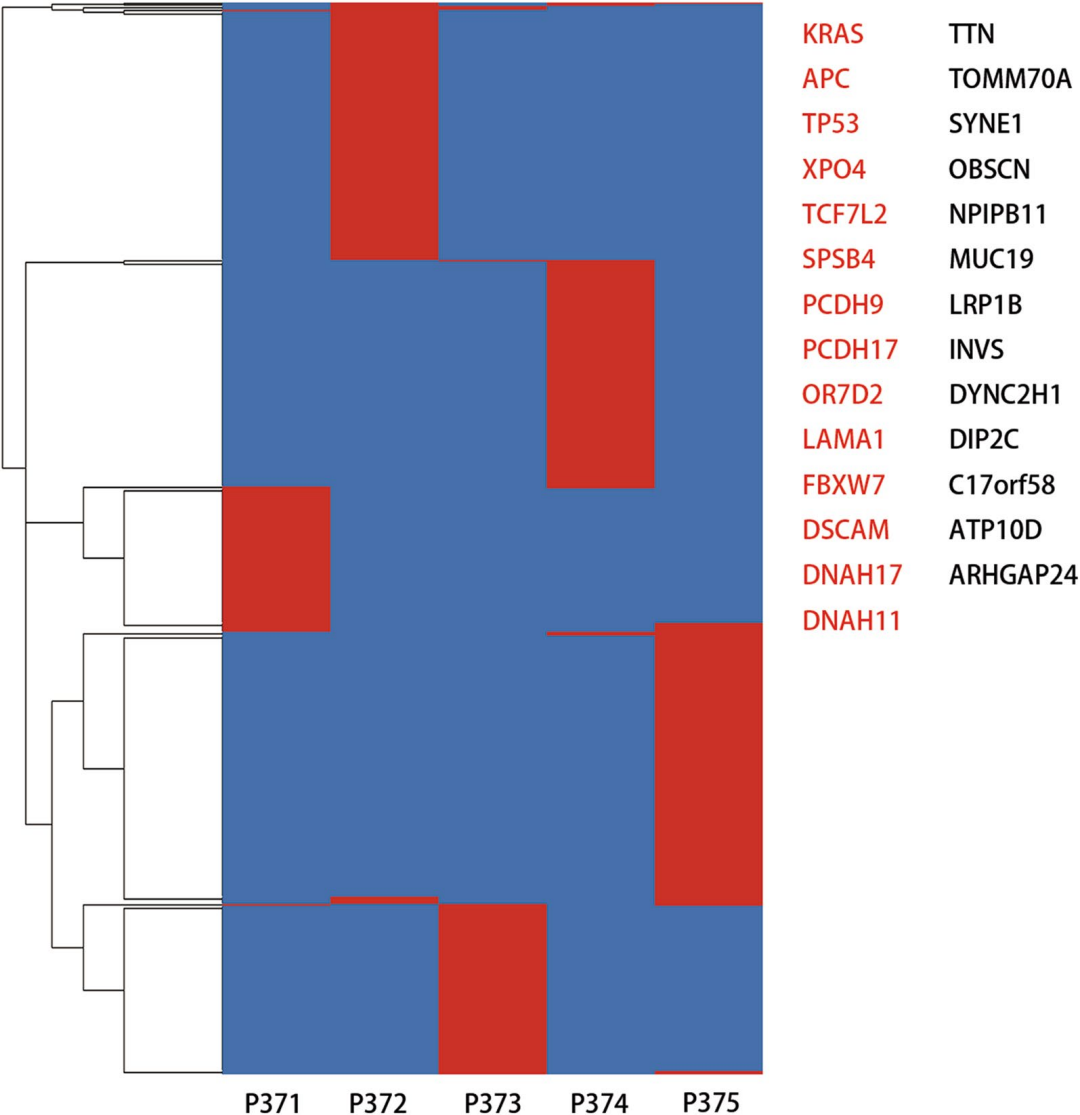
Potential genetic determinants of CRC LM. Heatmap showing the distribution of LM specific nonsynonymous SNVs. Presence (red) or absence (blue) of each mutation is marked for each tumor within one individual. Sample IDs are shown at the bottom of the heatmap. Mutated genes present in at least two LM lesions are listed on the right of the heatmap. Mutated genes present in both primary and metastatic CRC are marked in red

Next, we studied the SNVs that occurred in the LM lesions (Fig. 6). Mutations in KRAS, APC, TP53, XPO4, TCF7L2, SPSB4, PCDH9, PCDH17, LAMA1, FBXW7, ARHGAP24, ATP10D, C17orf58, DIP2C, DNAH17, DNAH11, DSCAM, DYNC2H1, INVS, LRP1B, MUC19, NPIPB11, OBSCN, OR7D2, SYNE1, TOMM70A, and TTN were identified in more than two samples, and additional SNVs were found in each individual patient. Mutations in KRAS, APC, TP53, XPO4, TCF7L2, SPSB4, PCDH9, PCDH17, OR7D2, LAMA1, FBXW7, and DNAH17 were also found in the primary tumors. Mutations in ATM, KIT, PIK3CA, and SMAD4 have frequently been found in CRC with synchronous LM, and FBXW7, SMO, and STK11 were frequently mutated in CRC with metachronous LM; CDKN2A, FGFR2, GNAS, JAK3, and SRC were mutated only in metachronous LM [37]. However, except FBXW7, other genes were rarely mutated in our samples.

Functional predictions of the nonsynonymous mutations in LM were then made. The top 10 GO terms based on biological processes, cellular component and molecular function are shown in Additional file 10: Figure S3A. The top two affected biological processes were homophilic cell adhesion via plasma membrane adhesion molecules and cell adhesion. The top two affected cellular components were M band and axoneme. The top two altered molecular functions were dynein intermediate chain binding and dynein light intermediate chain binding. We then performed KEGG analysis with genes mutated in LM. The top 20 pathways are displayed in Additional file 10: Figure S3B. Several of the affected pathways in CRC LM were cancer related. The PPI network of mutated genes in LM was also constructed (Additional file 11: Figure S4).

Genes mutated only in LM lesions include ARHGAP24, ATP10D, C17orf58, DIP2C, DNAH11, DSCAM, DYNC2H1, INVS, LRP1B, MUC19, NPIPB11, OBSCN, SYNE1, TOMM70A, and TTN. ARHGAP24 inhibits cell proliferation and cell cycle progression and induces apoptosis of lung cancer [38]. Silencing of ARHGAP24 activates the β-catenin signaling pathway to promote lung cancer cell migration and invasion [39]. DIP2C expression was found to be decreased in the basal-like and HER-2 breast cancer subtypes [40]. In a human cancer cell line, knockdown of DIP2C was found to induce extensive DNA methylation, gene expression variation, cell death, and epithelial–mesenchymal transition [41]. LRP1B has been reported to be down-regulated in colon cancer tissues, and impedes the proliferation, migration, and metastasis of colon cancer cells [42]. It is also often mutated in patients with melanoma and non-small-cell lung cancer. A higher tumor mutation burden was found in patients with LRP1B mutations [43]. Genomic meta-analysis has indicated that OBSCN plays an important role in GPCR, RAS, p75, and Wnt signaling. OBSCN likely regulates breast cancer progression and metastasis by interacting with many cancer genes [44]. SYNE1 polymorphism increases the risk of invasive epithelial ovarian cancer [45]. TOMM70A has been identified as a tumor suppressor gene in xenograft studies [46]. The functions of ATP10D, C17orf58, DSCAM, and INVS have not yet been reported.

## Discussion

In this study, an in-depth analysis of somatic mutations, somatic copy number alterations, and clonal structures revealed that the genomic alterations in primary and metastatic CRCs show various levels of discordance, indicating substantial levels of intertumor heterogeneity. Analysis of clonal evolution suggested that the founding clone in the primary tumor also evolved into the metastatic clone, regardless of whether the LM was detected synchronously or metachronously with the primary tumor. A few metastasis-specific mutations were identified, suggesting that essential mutations for LM might be pre-existing in primary tumors.

We observed substantial levels of intertumor heterogeneity in SCNAs and SNVs in primary CRCs and matched LMs. SCNAs often result in gene dosage effects that enhance tumor growth by up-regulating oncogenes or down-regulating tumor suppressors [47]. SCNAs were acquired by the primary tumor at the earliest stages during tumorigenesis and were inherited by metastatic tumors through tumor evolution. Trunk mutations were considered to be ‘early’ events because they were present in a large portion of cancer cells, had a relatively high mutation copy number, and occurred prior to the most recent clonal expansion [48]. Frequent mutations in cancer driver genes, including APC, TP53, and KRAS, promote colorectal tumorigenesis by modulating critical cellular pathways to achieve selective growth advantages for mutated cells [49]. Upon progression, primary and metastatic tumors acquired lesion-specific SCNAs and SNVs. The dynamic adaptation of tumors to local and distant organs is guaranteed by the myriad of cancer gene alterations [50]. Inter- and intra-tumor heterogeneity has also been reported in CRCs and their matched liver metastases [51].

Our clonal evolution analysis suggests that the founding clone of the primary tumor also evolved into the metastatic clone. This finding has important implications for the genetic prognosis of metastasis and the contribution of individual malignant clones to tumor progression should be investigated. LM-specific clones are not related to primary tumor-specific clones, which are formed by the transformation of normal lung cells. Multiregional WES of matched primary and metastatic lymph node tumors of CRC also revealed a polyclonal seeding of metastasis [52]. Through analysis of the spatial mutation categories and phylogenetic structures of primary CRCs and matched liver metastases, Kim et al. [51] identified branched evolutionary patterns in CRC genomes and suggested that preexisting subclones in primary lesions were responsible for the seeding of liver metastases. A similar evolutionary pattern has been proposed in acute myeloid leukemia relapse [53]. Naxerova et al. [54] found that two different lineage relationships between lymphatic and distant metastases exist in CRC; 65% of lymphatic and distant metastases were generated by independent subclones in the primary tumor, while 35% of cases shared a common subclonal origin. In contrast, Werner-Klein et al. [55] reported that early disseminated melanoma cells were genetically immature; they acquired critical alterations outside of the primary tumor, and thereby gained the ability to form a colony.

Comprehensive sequencing analyses of primary and metastatic cancer genomes have suggested that minimal additional genetic alterations are required for carcinoma cells to give rise to metastasis [56]. Accordingly, our study identified 27 mutated genes present in more than one metastatic sample, of which 14 were also found in primary tumors. These results suggest that the core mutations promoting cancer metastasis might already exist in primary cancer genomes. Indeed, Kim et al. [51] identified metastasis-clonal mutations in only 0.7% to 15.6% of total mutations in CRC liver metastases. In pancreatic cancer, it has been shown that new mutations acquired during metastatic development do not overlap across different patient samples [57]. Moreover, it has been shown that driver gene mutations not shared by all metastases are unlikely to have functional consequences [58]. Colon adenoma genomes were shown to be almost as old as invasive cancers, indicating that the time needed for metastatic formation is relatively short compared to the life span of CRC [51, 59].

## Conclusion

Our analysis of five CRC patients with synchronous or metachronous LM reveals discordant genomic alterations in primary and metastatic CRCs, indicating substantial levels of intertumor heterogeneity. Analysis of clonal evolution suggests that the founding clone in the primary tumor also evolved into the metastatic clone; mutations identified in primary tumors might be crucial for metastasis. These findings suggest that specific individual malignant clones contributing to cancer progression should be identified during the genetic prognosis of metastasis.

## Supplementary information

**Additional file 1: Table S1.** Clinicopathological characteristics of colorectal cancer patients with isolated initial lung metastasis.

**Additional file 2: Table S2.** Sequencing information of the five primary tumors, five metastatic tumors and five non-cancerous comparators.

**Additional file 3: Table S3.** Summary of synonymous and nonsynonymous somatic mutations identified in the five CRC patients with LM.

**Additional file 4: Table S4.** Significantly mutated genes in CRC patients with LM.

**Additional file 5: Table S5.** Somatic copy number variation and affected genes.

**Additional file 6: Figure S1.** Recurrent CNA and affected pathway analyses for the five CRC patients with LM. (A) Distribution of GISTIC regions in CRC as reported by TCGA. The colors represent copy number gain (red) and loss (blue). (B) KEGG significant enrichment analysis for high-risk genes localized in recurrent CNA regions. High-risk genes are defined as genes documented in KEGG pathways in cancer. The enrichment score indicated that the percentages of high-risk genes belonged to the corresponding pathway. The left y-axis represents the 11 enriched pathways. Bubble sizes indicate the number of high-risk genes in the corresponding pathway, and bubble colors represent the enrichment P value.

**Additional file 7: Table S6.** Significant copy number gains and losses.

**Additional file 8: Table S7.** KEGG analysis of high-risk genes.

**Additional file 9: Figure S2.** Clonal population structures in the five CRC patients with LM. The posterior distribution of cellular prevalence for each cluster is shown in different colors in the violin plots predicted by PyClone. The number of mutations in each cluster are shown in the horizontal axis. Sample names are labeled on the top of each violin plot.

**Additional file 10: Figure S3.** GO and KEGG analysis of genes mutated in LM. (A) GO enrichment analysis of genes mutated in LM. Bar charts showing the top 10 GO terms for molecular function, cellular process, and biological process ranked by enrichment score. (B) KEGG significant enrichment analysis for mutated genes localized in LM. The left y-axis represents the top 20 enriched pathways. Bubble sizes indicate the number of genes in the corresponding pathway, and bubble colors represent the enrichment P value.

**Additional file 11: Figure S4.** PPI net work of genes mutated in LM. The PPI network of genes mutated in LM was constructed using Cytoscape.

## Data Availability

The datasets during the current study available from the corresponding author on reasonable request.
