# Supplementary material for: Genomic profiling of colorectal cancer with isolated lung metastasis
Source: Cancer Cell Int. 2020 Jul 1;20:281. doi: 10.1186/s12935-020-01373-x (PMC7329491; doi:10.1186/s12935-020-01373-x)
Supplement: Supplementary file 1 — Additional file 1: Table S1. Clinicopathological characteristics of colorectal cancer patients with isolated initial lung metastasis. [file 12935_2020_1373_MOESM1_ESM.docx]

**Table S1 Clinicopathological characteristics of colorectal cancer patients with isolated initial lung metastasis**

| **ID** | **Gender** | **Age** | **Sample** | **Tumor** | **T stage** | **N stage** | **Location** | **Longest diameter(cm)** | **Type** | **Vascular thrombus** | **Treatment after primary tumor resection** |
| --- | --- | --- | --- | --- | --- | --- | --- | --- | --- | --- | --- |
| **P371** | Male | 53 | 371T | Primary | T3 | N0 | Ascending colon | 3.5 |  | N | None |
|  |  |  | 371LM | Metastatic |  |  | Left lung | 2.0 | Metachronous |  |  |
| **P372** | Female | 47 | 372T | Primary | T3 | N1 | Rectum | 3.5 |  | Y | Oxaliplatin |
|  |  |  | 372LM | Metastatic |  |  | Left lung | 2.7 | Metachronous |  | Capecitabine |
| **P373** | Male | 70 | 373T | Primary | T3 | N1 | Sigmoid colon | 5.0 |  | N | None |
|  |  |  | 373LM | Metastatic |  |  | Right lung | 4.1 | Synchronous |  |  |
| **P374** | Female | 67 | 374T | Primary | T3 | N0 | Rectum | 4.5 |  | N | Oxaliplatin |
|  |  |  | 374LM | Metastatic |  |  | Right lung | 1.2 | Metachronous |  | Capecitabine |
| **P375** | Male | 68 | 375T | Primary | T3 | N1 | Rectum | 3.5 |  | N | Oxaliplatin |
|  |  |  | 375LM | Metastatic |  |  | Right lung | 1.5 | Synchronous |  | Capecitabine |

All the five primary tumors were adenocarcinoma, moderately differentiated, and microsatellite stable (MSS).
