# Supplementary material for: Genomic profiling of colorectal cancer with isolated lung metastasis
Source: Cancer Cell Int. 2020 Jul 1;20:281. doi: 10.1186/s12935-020-01373-x (PMC7329491; doi:10.1186/s12935-020-01373-x)
Supplement: Supplementary file 2 — Additional file 2: Table S2. Sequencing information of the five primary tumors, five metastatic tumors and five non-cancerous comparators. [file 12935_2020_1373_MOESM2_ESM.docx]

**Table S2 Sequencing information of the five primary tumors, five metastatic tumors and five non-cancerous comparators**

| **Sample ID** | **On target > 10X coverage** | **On target > 50X coverage** | **On target mean coverage** |
| --- | --- | --- | --- |
| 371T | 91.66% | 86.09% | 237.28X |
| 371LM | 91.06% | 84.65% | 214.09X |
| 371N | 91.30% | 85.26% | 223.23X |
| 372T | 91.58% | 86.23% | 237.89X |
| 372LM | 91.44% | 85.93% | 229.31X |
| 372N | 91.43% | 85.78% | 225.62X |
| 373T | 91.82% | 86.11% | 219.00X |
| 373LM | 91.60% | 85.94% | 236.24X |
| 373N | 91.84% | 86.41% | 231.08X |
| 374T | 91.98% | 89.19% | 457.51X |
| 374LM | 91.82% | 88.57% | 405.02X |
| 374N | 91.50% | 85.80% | 223.62X |
| 375T | 91.69% | 88.55% | 474.87X |
| 375LM | 91.35% | 85.16% | 226.04X |
| 375N | 91.86% | 88.81% | 455.64X |
